# KMD-Enhanced MALDI-ToF MS for Polymer Biodegradation Analysis

**DOI:** 10.1021/jasms.6c00101

**Published:** 2026-07-14

**Authors:** Sophia Y. van Mourik, Heather J. Walker, Anthony J. Ryan

**Affiliations:** † Dainton Building, 7315 The University of Sheffield, Sheffield S3 7HF, U.K.; ‡ biOMICS Mass Spectrometry Facility, The University of Sheffield, Sheffield S10 2TN, U.K.

**Keywords:** MALDI-ToF MS, Kendrick Mass Defect Analysis, Polymer Degradation Mechanisms, Biodegradation, Oligomer Characterization

## Abstract

A novel and tailored mass spectrometry workflow is presented for the analysis of polymer biodegradation products, integrating principles from the ‘omics’ sciences to address the challenges of complex mixture characterization. The workflow combines methodologies from polymer analysis and metabolomics, offering a comprehensive approach to data acquisition and processing, with practical considerations for user implementation. Central to the analytical strategy is the application of Kendrick Mass Defect (KMD) theory, which enhances spectral visualization and facilitates the identification of biodegradation products and metabolites within heterogeneous samples. By combining accessible and open software tools along with custom R scripts, the workflow supports reproducible MS data analysis, providing a platform for advancing polymer biodegradation research. Biodegraded samples, due to their complex polymer and metabolite content, typically generate noisy spectra, which require intensive filtering to distinguish polymer fragments from microbial metabolites. While pure polymer spectra are generally cleaner, they still necessitate specific peak detection methods, emphasizing the need for tailored data processing protocols.

## Graphical abstract

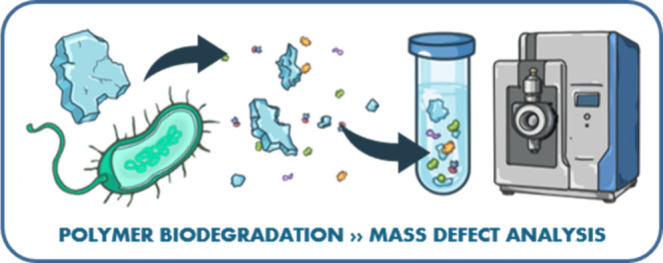

## Introduction

The high specificity and relatively mild reaction conditions typically associated with biological degradation systems, make it an attractive, alternative process for polymer remediation and breakdown of difficult to process materials and feed streams which would otherwise be processed using mechanical or chemical methods. Furthermore, uncovering the mechanisms behind biodegradation of polymeric materials can reveal how synthetic materials interact with the environment.

A fundamental challenge in this mechanistic understanding is that the biodegradation supernatant is a complex mixture of molecules of compromising polymer fragments and biological metabolites. This is particularly the case when dealing with mixed polymer feedstocks. Elucidating mixture composition and subsequent mechanisms from mass spectrometry analysis becomes exceedingly difficult in highly convoluted systems.

Biodegradation is characterized by the bulk material deterioration, culminating in the assimilation and conversion of polymer carbon backbones into *CO*_2_, a process measurable through gas evolution monitoring.^1,2^ In contrast polymer industry prioritizes the preservation of physical properties to maintain long-term durability and functionality, as any slight changes in these properties can indicate degradation, whether biological or otherwise.

Analytical techniques in relation to degradation analysis can broadly be classified as those assessing bulk property changes versus those probing molecular-level transformations.^3^

Bulk material properties are frequently evaluated through characterization of polymer morphology alongside measurements of crystallinity and melting transitions, as these parameters serve as key indicators of the preservation or loss of desirable mechanical performance. Gravimetric analysis is commonly employed for this purpose, providing a straightforward quantitative assessment of polymer deterioration via changes in mass. In parallel, examinations of surface topography and wettability offer additional insight, as alterations at the interface can be correlated with microbial colonization and the propensity for biofilm formation.^4,5^

Analytical techniques in relation to degradation analysis can also be categorized according to their ability to interrogate molecular changes. Such macroscopic property shifts typically arise from underlying molecular-level transformations, necessitating spectroscopic approaches to elucidate degradation pathways. Variations in molar mass distribution, the emergence or loss of functional groups, and the formation of degradation products can be assessed to inspect these molecular changes. Both Fourier Transform Infrared Spectroscopy (FTIR) and Nuclear Magnetic Resonance (NMR) spectroscopy, enable sensitive detection and quantification of polymer chemical changes.^6,7^ For cross-linked materials the transition from large networks to oligomeric and monomeric units is a crucial step in the fragmentation of the polymer material and is conventionally confirmed by Size-Exclusion Chromatography (SEC) as it tracks the reduction in molecular weight as the backbone fragments. Mass Spectrometry (MS) methods such as Matrix-Assisted Laser Desorption/Ionization Time-of-Flight MS (MALDI-ToF MS) are crucial for elucidating small molecules, thereby uncovering monomer assimilation and mineralization pathways during biodegradation.

The inherent complexity of biological matrices, particularly the presence of biological contamination in samples like biodegraded polymer supernatants, introduces significant limitations to conventional polymer analysis workflows.

The successful application of MALDI-TOF MS to synthetic polymers necessitates rigorous preanalytical ″cleaning,″ involving extensive physical separation, extraction, and filtration to mitigate ionization suppression and spectral convolution. A fundamental bottleneck remains the ionization efficiency, which is governed by a multifaceted interplay of analyte-matrix solubility,^8^ cationization reagents, and instrumental dynamics.^9^

Specifically, unlike small molecules, synthetic polymers typically require precise cationization (e.g., Na^+^ or Ag^+^ salts) to facilitate ion attachment. This process is highly sensitive to the polymer’s end-group chemistry, polarity, and structural topologies and the polymer and degradation products.^10^ Consequently, traditional MALDI-TOF MS requires a well-defined, high-purity analyte system to produce interpretable data. This requirement presents a significant barrier for biodegraded samples; the inherent chemical heterogeneity and ill-defined breakdown products create a ″noisy″ matrix that effectively precludes the definitive characterization.

To address the limitations imposed by conventional sample preparation, this article outlines the development and application of a novel workflow that bypasses conventional preanalytical constraints by analyzing raw biodegradation supernatants through a computational lens. While direct analysis yields highly complex spectra, this challenge is mitigated by a pipeline that leverages the Kendrick Mass Defect (KMD) principle.

The Kendrick mass is a mathematical transformation that redefines the mass scale by setting a chosen structural unit, such as the CH_2_ group, to an exact integer value.^11^ Unlike the standard IUPAC scale, which defines the ^12^C isotope at exactly 12 u, the Kendrick scale rescales the repeating unit (e.g., 14.0269 for CH_2_) to an integer (14.00). Consequently, this shift ensures that all molecules within a homologous series share the same fractional mass. The techniques exploits small mass differences which enable the visual isolation of polymer families from complex biological backgrounds as well as the discernment of structural variations (e.g., functional groups or chain branching).^12^ By utilizing postanalytical sorting, the method organizes detected fragments, isolates ion series and filters biological noise to allow the detection of both small bacterial metabolites and polymeric substances.

Crucially, KMD relies solely on the accurate mass of the spectral peak, making it intensity independent. Being inherently independent of ionization efficiency and peak intensity, the KMD filter offers two critical analytical benefits. First, by depending solely on the mass of the spectral peak, it ensures that low-intensity products in the sample spectra, which might be filtered out during traditional data processing, are retained. Second and more importantly, this mass-based approach effectively compensates for the inherent small mass ionization bias prevalent in MS,^10^ especially in techniques like MALDI-ToF MS. In these methods, the low ionization efficiencies typically observed for larger molecules can lead to vital high-mass degradation components being masked, but the KMD-based method analysis ensures the comprehensive detection of these essential species.

Beyond structural sorting, the workflow is designed to provide versatile data outputs that support expanded peak and sample analysis. This includes the exporting of Total Ion Count (TIC) tables, enabling integration with online resources like MetaboAnalyst,^13^ as well as enabling further multivariate statistical assessments such as Principal Component Analysis (PCA). This approach retains full biological context while overcoming challenges related to variable ionization efficiency, thereby maximizing the information extracted from the complex polymer degradation mixture.

## Methodology and Workflow

Each step of the workflow, from raw data acquisition to downstream interpretation, is described in detail in the following section. Particular attention is paid to methodological considerations, software tools, and data handling strategies that underpin the analytical pipeline. While the workflow is tailored and may require iterative refinement by the user, this article aims to provide a clear and practical guide to support its implementation. Figure 1 illustrates a structured overview of the workflow that has been developed, which is supported by a suite of custom R scripts^14^ and openly accessible software. As a demonstration this article will walk through the analysis of a water-soluble mPEG compound that has undergone biodegradation.

**Figure 1:**
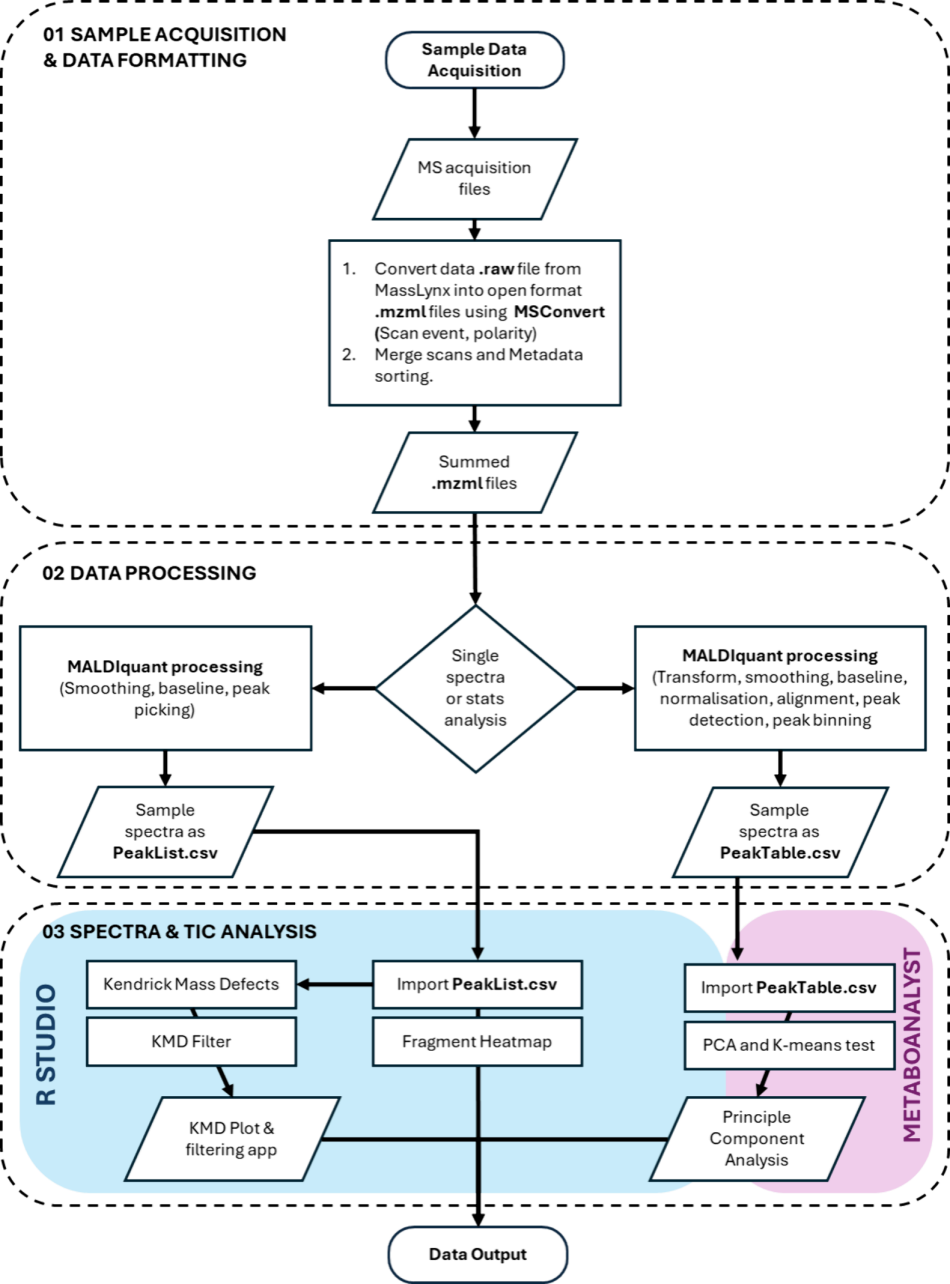
Process diagram illustrating the data processing workflow developed for MS analysis, with each step executed via a corresponding R script. (1) MALDI ToF MS data acquisition of biodegradation experiment samples to generate .raw files, which are converted to. mzML format using MSConvert, then merged and summed in RStudio using the MALDIquant package. (2) Resulting data undergoes standard preprocessing, including spectral normalization and peak detection, producing PeakList.csv and PeakTable.csv outputs. (3) Downstream analysis either in RStudio for Kendrick Mass Defect calculations or via MetaboAnalyst, an open-source metabolomics platform.

## Sample Acquisition and Data Formatting (Pre-Processing)

### Biodegradation Experiments and Sample Preparation

Three distinct sample groups were evaluated in this study: untreated (mPEG-A), control (mPEG-C), and degradation products (mPEG-D). Preparation protocols for each are detailed below:

- Untreated Samples (mPEG-A): Methoxy-polyethylene glycol [CH_3_(O–CH_2_CH_2_)_n_–OH] (mPEG; Sigma-Aldrich) was dissolved in tetrahydrofuran (THF) at a concentration of 1 mg/mL.
- Control and Degradation Samples (mPEG-C and mPEG-D): The mPEG powder was dissolved in Bushnell-Haas (BH) growth medium at a concentration of 1 mg/mL.

For the degradation experiment, samples were inoculated with a 1 mL aliquot of a specialized microbial consortium. This community was cultured following the established protocols described by van Mourik.^15^ Following inoculation, all treated (mPEG-C and mPEG-D) vessels were incubated at 67 °C for a duration of 2 days to facilitate microbial action.

Samples were characterized via MALDI-ToF MS using a Waters G2 Synapt mass spectrometer. Supernatants and untreated controls were mixed directly with a 2-(4-Hydroxyphenylazo) benzoic acid (HABA) matrix (5 mg/mL in methanol with 0.1% TFA) before spotting 1ul directly onto a MALDI target plate. HABA was selected based on its reproducible spot morphology and good ionization performance for the target oligomeric/polymeric species in complex aqueous sample matrices.

Data were acquired in positive ionization mode over 180 s with laser energy of 320 and 1000 Hz firing rate. These parameters, particularly those for ionizing polymer fragments above 3000 Da, were refined through optimization across sample sets. The calibration was performed using red phosphorus dissolved in acetone at a calibration range of 100–1500m/z or 1500–500m/z.

Due to variability in sample composition, arising from the incorporation of biological low-molecular-weight components into the polymer systems, sample preparation conditions were iteratively optimized to achieve reproducible spot morphology and adequate ionization. To this end, the analyte-to-matrix ratio was refined, with a 1:1:1 (v/v/v) ratio of supernatant:methanol:matrix masterbatch established for untreated samples (mPEG-A), while the experimental samples (mPEG-C and D) required adjustment to a 1:2:2 ratio. The elevated water content and additional inorganic salts from the Bushnell–Haas medium; lowered water activity and disrupted uniform matrix–analyte cocrystallization, resulting in heterogeneous spot morphology and reduced ionization efficiency in the treated sample. Increasing the proportions of methanol and matrix counteracted these effects by improving evaporation dynamics and diluting the ionic load.

## Data Processing

### MSConvert and Merging Raw Data Scans

To convert scans to open format for processing in R studio and the subsequent pipeline, MS data files from the instrument software, need to be converted into.mzmL files which can be done via free software called MSConvert.^17^

### R Script Normalization and Processing

Spectral processing was conducted using open-source R packages including MALDIquant, *MALDIquantForeign, MnSBase* (via *BiocManager* 3.20), and *dplyr*. Sample data were processed using an adapted preprocessing and reduction script from Parker et al.^18^ with RStudio (versions R.4.4.2 and R2024) employed to convert raw.mzML spectra into a combined Total Ion Count (TIC) sheet for downstream statistical analysis. The choice of output depends on the intended data analysis. A single-spectrum peak list can be generated for detailed inspection of individual samples, particularly useful for KMD analysis and structural characterization of degradation fragments. Alternatively, a peak table, commonly used in metabolomics, compiles binned peaks across all samples to enable multivariate analysis and assess repeatability, variability, and broader data set trends.

Default processing settings recommended in *BiocManager* packages are optimized for small-molecule metabolite detection, often employing excessively high resolution for polymers and oligomers that span a much larger *m*/*z* range. This mismatch can impair peak detection accuracy, making it essential to adjust resolution and detection thresholds for reliable identification of polymeric fragments in mixed samples.

Data processing steps included normalizing the spectra, followed by peak alignment and binning (single *m*/*z* column truncation) to prepare the intensity data for linear regression analysis. Data normalization in this workflow comprises four key steps: transformation, smoothing, baseline correction, and spectra calibration (including peak alignment, picking, and binning). Each step plays a critical role in preparing spectral data for accurate analysis. The optimal method for each is determined empirically by evaluating output quality and selecting the approach that provides the most effective normalization for the specific data set. Final settings are evaluated using LINEST and R2. This data processing is performed in accordance with the van Mourik method^15^ and described in the Supporting Information.

## Kendrick Mass Defect Analysis and Tic Table Generation

### Kendrick Mass Defect Theory

Observed *m*/*z* values from MS spectra (Figure 2A) are mathematically transformed using eqs 1 and ([Disp-formula eq2]) to calculate the Kendrick Mass (KM) and Kendrick Mass Defect (KMD), respectively, based on the repeat unit mass of poly­(ethylene oxide) (C_2_H_4_O, 44/44.026215 Da = 0.999404).

1 $$\mathrm{Kendrick Mass}=\left(\mathrm{observed}m/z\right)\times \left(\frac{\mathrm{Nominal base unit mass}}{\mathrm{IUPAC base unit mass}}\right)$$

2 Kendrick
Mass Defect⁡(KMD)=round(KM)−KM

**Figure 2:**
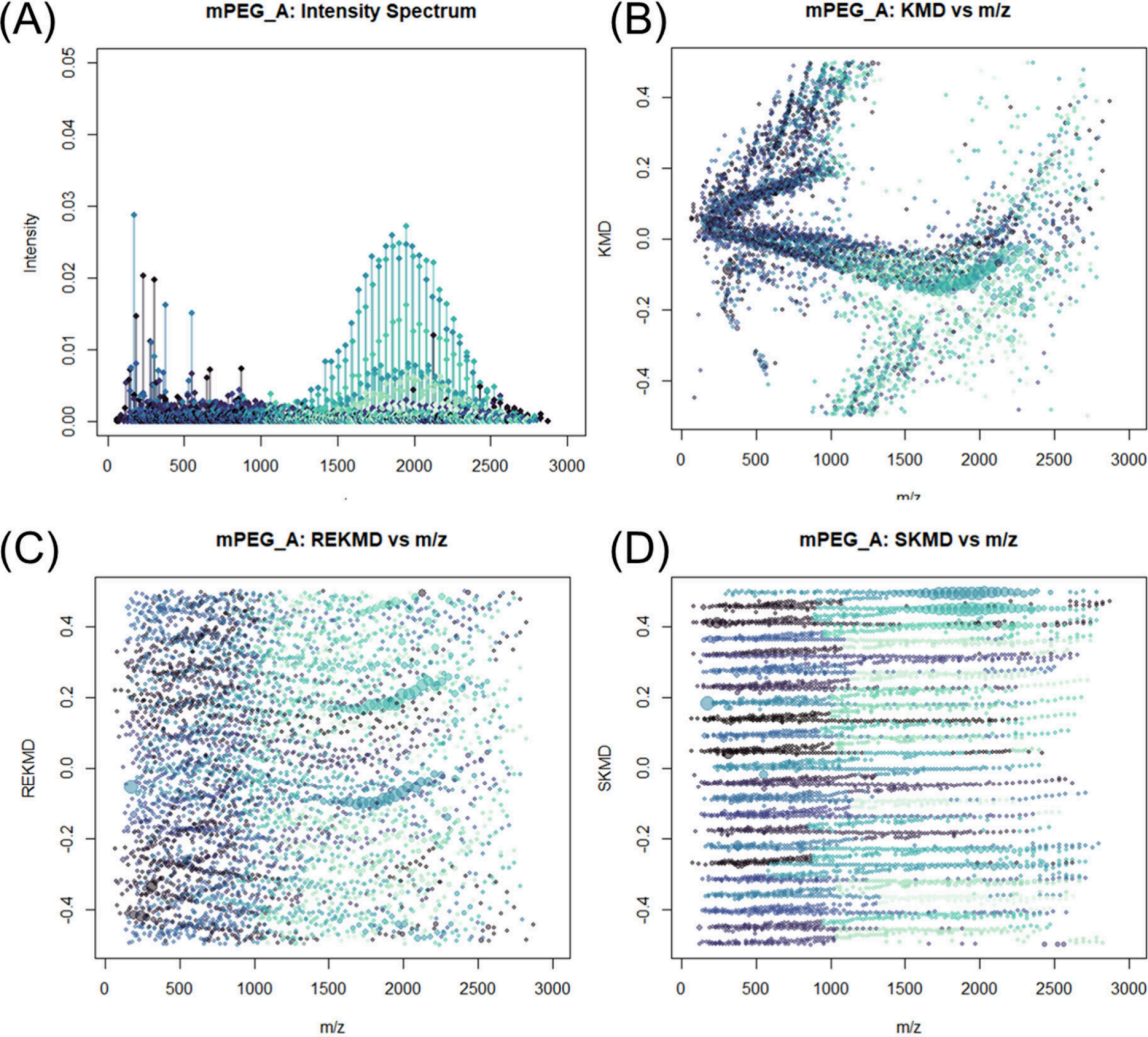
Color-based group assignment and multimodal visualization of the mPEG-A control. (A) Original mass spectrum showing peak intensity vs *m*/*z*. (B) Traditional Kendrick Mass Defect (KMD) plot demonstrating the clustering of mPEG species based on the repeating unit. (C) Resolution-enhanced KMD (REKMD, R = 44/43) and (D) Scaled KMD (SKMD, R = 1/44) plots utilizing a mass-to-charge (*m*/*z*) alignment tolerance of 0.02 Da. Grouping was performed via an automated R script to facilitate color-coding.

Plotting the calculated KMD against the nominal Kendrick mass aligns ion species as seen by the major curved cluster in Figure 2B. The observed clustering confirms the characteristic repeating unit of poly­(ethylene oxide) (PEO), identifying all detected species as derivatives of the PEO backbone.

This visual clustering effectively isolates the polymer backbone from noise. Inspecting the plot further a clear splitting of the data based on its trend (line/gradient) and deviation reveals the presence of at least two separate PEO-containing species and a third fragmented species at a lower molecular mass range.

In the low mass region (0 *to* 1500 *m*/*z*)), a straight-line cluster with a slight positive gradient is observed. This cluster is attributed to the mPEG homopolymer chains between 1 and 44 repeats, representing the trailing distribution of *n*-mers. Above 1500 *m*/*z*, shows a higher crowding of points, attributed to the bulk of the mPEG ion *n*-mer peaks, corresponding to the major peaks seen in the MS spectrum (Figure 2A). This curved tailing in the mass region greater than 1500 *m*/*z* is a combination result of calibration-related effects and intrinsic structural heterogeneity within the oligomer distributions.

While effective for simple spectra, standard KMD plots (Figure 2B) lack the resolution to decipher the complex mixtures typical of polymer biodegradation. In these systems, the overlap of multiple ion species is compounded by the need to retain low-intensity peaks essential in detecting metabolites. Consequently, a basic KMD plot becomes insufficient for detailed identification, functioning only as a broad grouping tool.

### Resolution Enhanced Kendrick Mass Defect

Resolution-enhanced Kendrick mass defect (REKMD) introduces a fractional base unit (R/X) into the KMD calculation (eq 3 and ([Disp-formula eq4])) to rescale the KMD axis and address the limitations of traditional KMD.^19,20^ The fractional base unit effectively expands the mass defect space along the *y*-axis of the plot, which significantly increases the resolution and enables a clearer distinction of closely related ion species as shown in Figure 2C.

3 $$\mathrm{Resolution‐Enhanced KM}\left(\mathrm{REKM}\right)=\left(\mathrm{observed}m/z\right)\times \left(\frac{\mathrm{round}\left(\frac{R}{X}\right)}{\left(\frac{R}{X}\right)}\right)$$

4 REKMD=round(REKM)−REKM

This divisor (*X*) is typically chosen within the range of 2/3*R* to 2*R*, where *R* is the mass of the repeating moiety. For example, in the case of poly­(ethylene oxide) (PEO), *R* = 44, divisors between 30 and 88 would be typically chosen. Manually testing every divisor (e.g., generating 58 individual KMD plots for ethylene oxide) is time-consuming, and the number of possible divisors increases with larger moieties.

To enable more intuitive selection of the X divisor for the best separation for mass-spectral data, Nakamura et al.^21^ introduced the RANK1 and RANK2 functions which are graphical methods ranking separation power via plotted RANK minimums.

The selection of divisors 33 and 57, guided by the RANK1 method identified minima (Figure S7) that successfully separates clusters previously unresolved in standard KMD plots (Figure 3). Specifically, divisor 33 (Figure 3A) resolves the data into four distinct series, highlighting the carbon isotope separation of the mPEG backbone, arising from the natural abundance of ^12^C and ^13^C isotopes. Further refinement is seen with divisor 57 (Figure 3B), which reveals the separation of varying ion adducts, inferred visually by the larger data points representing higher-intensity peaks [M + Na]+ and [M + K]+ ion adducts and the smaller intensity species ([M + HABA]+, [M + HABA – H_2_O]+) which were previously not discernible.

**Figure 3:**
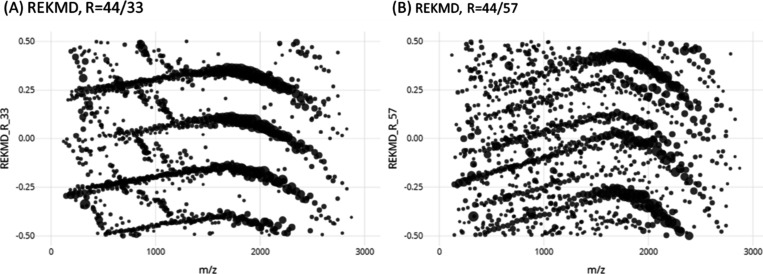
REKMD plots of the first two minima 33 and 57 selected from RANK1 function divisor plot. (A) With a divisor of *X* = 33, the initial KMD cluster is resolved into four distinct clusters, each maintaining the characteristic KMD plot appearance. (B) A divisor of *X* = 57 demonstrates superior separation power compared to *X* = 33, providing a clearer distinction between the data series.

However, even with the use of the RANK1 function to identify optimal divisor values, REKMD still introduces mathematical aliasing. This arises from scaling-induced compression of the mass defect space (*y*-axis), whereby distinct ion series are mapped onto the same apparent positions, reducing scatter and potentially obscuring underlying structural variability. At higher scaling factors, this effect approaches the compression limit, where excessive aliasing leads to significant overlap of ion series and a reduced ability to resolve genuine structural differences, particularly at *m*/*z* values above 1500.

### Scaled Kendrick Mass Defect

To overcome the compression limit and associated aliasing inherent in REKMD, thereby enabling more robust and automated graphical interpretation of mass defect data, the final step in this workflow utilizes the generalized KMD plots. Alton et al. applied a KMD adaptation to atmospheric data, but faced a similar complexity challenge where no single chemical unit could represent the diverse composition.^22^ To address this, they introduced an inverse integer scalar, mirroring the REKMD scalar. The mathematically equivalent replacement of *R*/*X* with *X*/*R* is shown in eq 5 and ([Disp-formula eq6]). This replacement facilitated zero-slope alignment based on elemental composition, though with reduced separation power compared to REKMD divisors.

5 $$\mathrm{Scaled KM}\left(\mathrm{SKM}\right)=\left(\mathrm{observed}m/z\right)\times \left(\frac{X}{R}\right)$$

6 SKMD=round(SKM)−SKM

In this generalized form *X* is a new integer scalar between 1 and 10 and this allowed for the elemental discernment of atmospheric gases. Given the small molecules present in biodegradation mixtures, this method is relevant for highly complex polymeric systems complicated by biodegradation processes. Applying this generalized KMD method to the biodegraded polymeric system described here allows for the utilization of the tool as a novel filtering and picking method.

This work specifically utilizes the computational rotation of the data, which forces the target ion series to align with a zero-slope, and in-turn automate assignment of homologous series via an R script based on the *x* = 0 line equations and a user defined mass defect threshold. The mathematical comparison of mass defect space and benefit of the zero-slope alignment of points is demonstrated in Figure 4.

**Figure 4:**
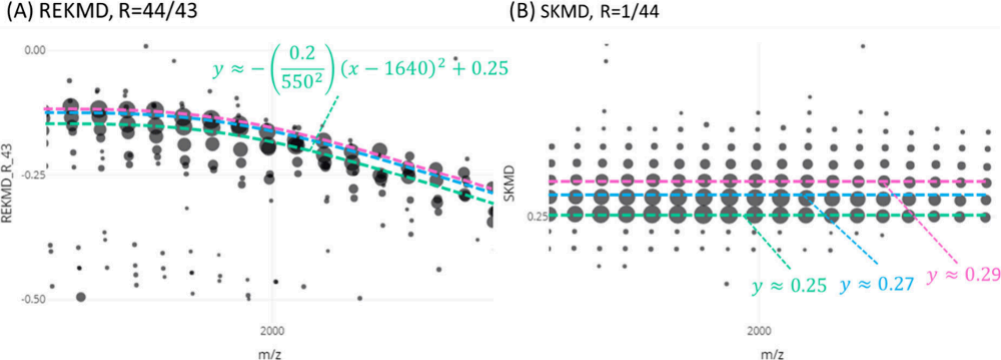
REKMD and SKMD plots: comparison of ion alignment. These plots compare the alignment of chemically related ions using REKMD and SKMD divisors. (A) REKMD points exhibit a more complex, shallow parabolic trend. (B) SKMD points align along a nearly horizontal straight line. Separated series that can be estimated by horizontal straight lines (*y* = *mx* + *C*), with *C* values of 0.25, 0.27, and 0.29, facilitating point selection based on SKMD values.

This SKMD approach provides greater flexibility to explore a wider range of scaling factors beyond the limitations of conventional REKMD visualization and is particularly effective for interrogating complex oligomeric distributions while enhancing visibility of subtle compositional trends across different repeat-unit relationships.

This autoassignment process then groups, and subsequently backward assigns and colors the KMD plots as well as the initial MS spectrum, as shown in overall workflow demonstration in Figure 2. The number of groupings generated within a sample is directly determined by a user-defined threshold, allowing the user to control the granularity of the analysis. The relationship between the threshold and the number of groups is inverse: a smaller threshold will generate a greater number of groups (by being more sensitive to minor variations), whereas a larger threshold will generate a smaller number of groups (by aggregating more species). This functionality allows the user to strategically tune the analysis to either focus on subtle chemical differences or on major polymeric species.

### Ion Adducts and Heatmap of Potential Chemical Species

In parallel to the KMD filter, the user should generate a fragment table based on their understanding of the system. This provides a list of potential ions to look for. Large metabolites, such as those identified in the preceding KMD analysis, are defined as partially degraded polymers or oligomers exceeding 500 Da in molecular weight. Compared to the original polymer, they exhibit a reduced molecular mass and increased polydispersity, a consequence of random chain scission. In contrast, small metabolites are single-peak compounds with a molecular weight below 500 Da, released from the larger oligomer chains or generated as byproducts of bacterial metabolism.

Theoretical masses of known and potential compounds, as well as their adducts, were calculated via a custom R script. For polymeric species, ‘M’ represents the mass of a single peak or a specific *n*-mer within the oligomeric distribution. All adduct masses were then determined based on the mass of the corresponding ion.

## Results and Discussion

### Kendrick Mass Analysis of Poly­(Ethylene Oxide) Mono-Methyl Ether

By inspecting the MS spectra (Figure 5) alongside the corresponding REKMD and SKMD plots as shown in Figure 2, we can identify the chemical nature of the groupings. This crucial step allows us to distinguish if a group represents a major polymeric species (a dominant, repeating molecule in the sample) or if it is just noise from a few small molecules.

**Figure 5:**
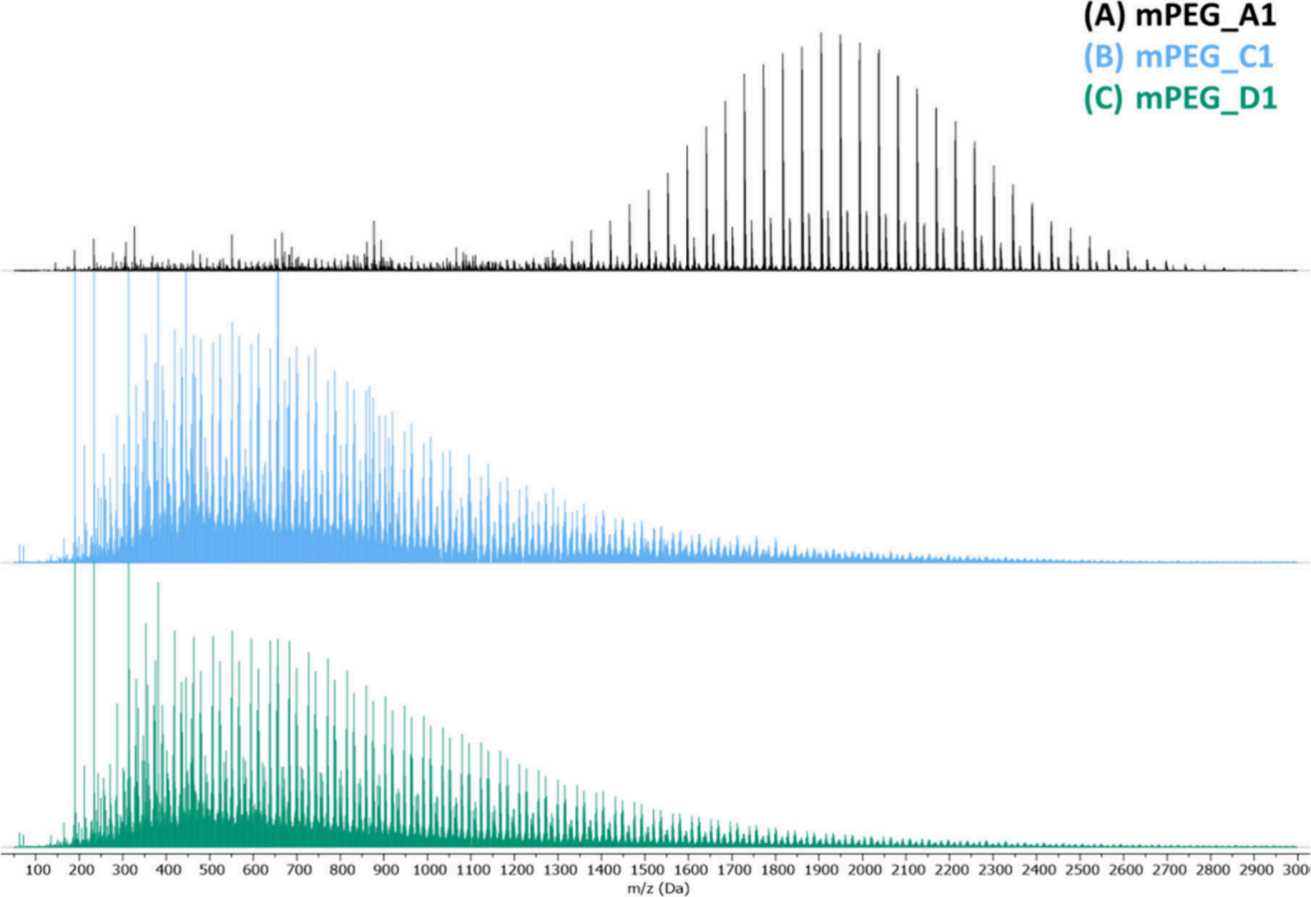
MALDI-TOF MS spectra illustrating the degradation of mPEG incubated in the presence with and without the biological culture. (A) Untreated solution mPEG in THF (mPEG-A), displaying two primary distributions centered at 2000 *m*/*z*, corresponding to [M + Na]^+^ and [M + K]^+^ adducts with a clear 44 Da repeat unit. (B) Abiotic blank (mPEG-C) incubated at 67 °C and pH 7, showing significant fragmentation and a shift toward lower molecular weight; the high density of overlapping oligomers and complex adduct formation obscure the original periodicity. (C) Inoculated mPEG (mPEG-D) under identical conditions, exhibiting a degradation profile.

Applying the KMD filter, Figure 6 illustrates the control mPEG-A sample partitioned into 33 discrete SKMD groupings. These groupings are sample-specific and generated through an iterative process that increments until all spectral data points are allocated. By systematically partitioning the data, the filtering tool enables granular analysis through the selection and inspection of specific groupings. As demonstrated at 33-, 5-, and 2-group resolutions in Figure 6, this iterative refinement overcomes overwhelming data complexity. Consequently, the approach transforms a dense, overlapping data set into a navigable collection of discrete chemical families, allowing for the precise identification of low-abundance species that would otherwise be obscured.

**Figure 6:**
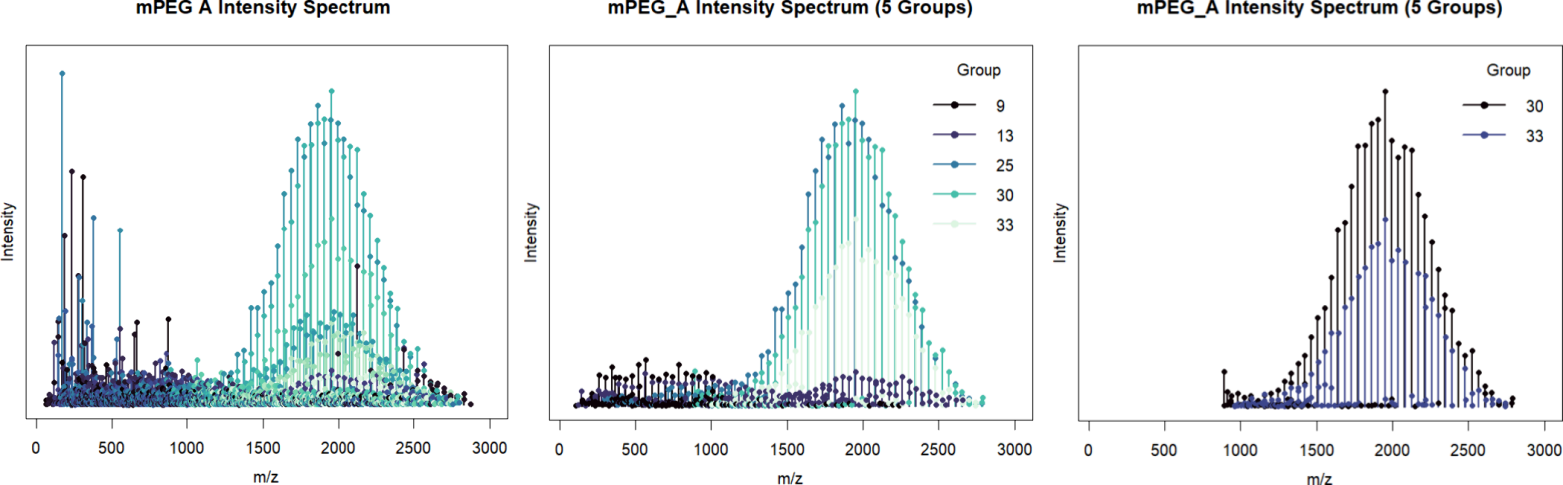
Kendrick mass defect group filtered MALDI-TOF MS intensity spectra for the mPEG-A control sample at varying group resolutions.

Figure 7 presents a detailed analysis of the control mPEG-A sample, where isolated SKMD groupings are assigned to three major adduct species. Notably, all three species display characteristic 44 *m*/*z* repeat units, a signature periodic spacing indicative of PEO monomeric fragments. Using cross referencing with adduct tables, group 9 is confirmed as the [mPEG+Na] ion adduct series and is centered around 2000 *m*/*z*, consistent with the expected profile of the analyzed mPEG polymer. Groups 29 and 30 were identified as polyethylene glycol (PEO) series, specifically the [PEO + H + HABA – H_2_O] and [PEO + K – H_2_O] ion adducts. Both groups are present at low intensity, often near the noise level. Their presence is most likely due to the initiation of EO polymerization by adventitious water, during industrial synthesis of the methanol-initiated mPEG polymer, yielding low *M*_w_ dihydroxy PEO, i.e. PEG.

**Figure 7:**
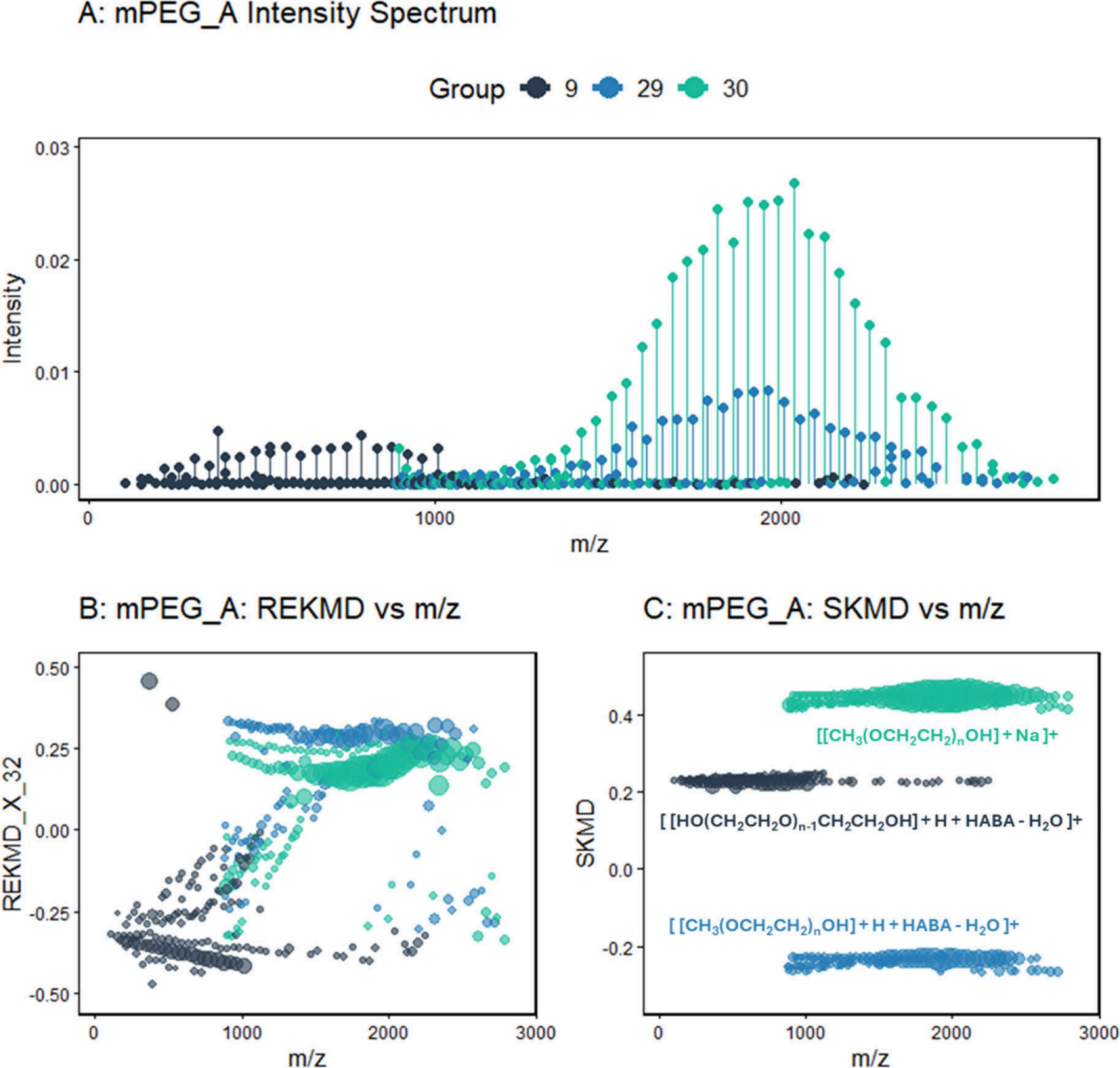
PEO-based peak series groupings via REKMD and SKMD plots of mPEG-A spectra. (A) MS Spectra of ion peaks indicating the colored series represented in subsequent REKMD and SKMD plots. (B) REKMD (*X* = 34) plot showing the alignment of 44 Da spaced peaks; consistent gradients indicate related polymer series. (C) SKMD (*X* = 1) plot, which clusters the same data points more distinctly along the *y*-axis. This horizontal gradient served as the basis for the automated grouping script. Colors in both plots correspond to SKMD group assignments, maintaining consistency across the *x*-axis (*m*/*z*).

attributable to residual ethylene oxide (EO) precursor remaining from the synthesis of the main mPEG polymer.

The structural heterogeneity of the identified species, specifically the adduct specific mass defect variation between alkali metal adduction (Na^+^) and matrix-derived fragments (HABA) manifests as distinct curved gradients in REKMD space (Figure 7B) at different y-intercepts. The observed curvatures reflect cumulative mass defects, where the unique signatures of specific end-groups and structural variations diverge increasingly from the PEO-repeating backbone as the degree of polymerization rises. By applying SKMD, these structural offsets are normalized, allowing for the clear horizontal partitioning of species that would otherwise overlap or blur in a standard intensity spectrum. Figure 7 highlights a pronounced structural heterogeneity brought on by the mPEG degradation, where SKMD effectively resolves the separation between alkali metal adducts and matrix-derived ions.

Following inoculation, the mPEG polymer distribution exhibits a pronounced shift toward the lower *m*/*z* region, accompanied by a transition from the original Gaussian-like profile to a markedly asymmetric, long-tailed distribution. A similar pattern is observed in the Bushnell–Haas (BH) medium controls, where the PEG already appears substantially degraded indicated by the Mw shift, consistent with predominant chain scission and hydrolysis processes. The degraded PEG profile in the BH control samples can most plausibly be attributed to steam sterilization step, as polyethylene glycol is known to undergo thermo-oxidative and hydrolytic chain scission under autoclave conditions.^16^

While all identified series remain ion adducts of mPEG, they represent truncated chain lengths relative to the untreated sample. Cross-referencing adduct tables identifies the primary distribution (Group 10) as [mPEG + H + HABA – H_2_O]^+^ , followed by [mPEG + Na – H_2_O]^+^ . Groups 1 and 32 correspond to the [mPEG+2K+H] + ion series; notably, PEO species were not detected among the major series, suggesting either their complete degradation or obscuration by spectral noise. This morphological change between the untreated (Figure 7) and ‘inoculated’ (Figure 8) samples, indicates chain degradation, characterized by a reduction in weight-average molecular weight (*M*_w_).

**Figure 8:**
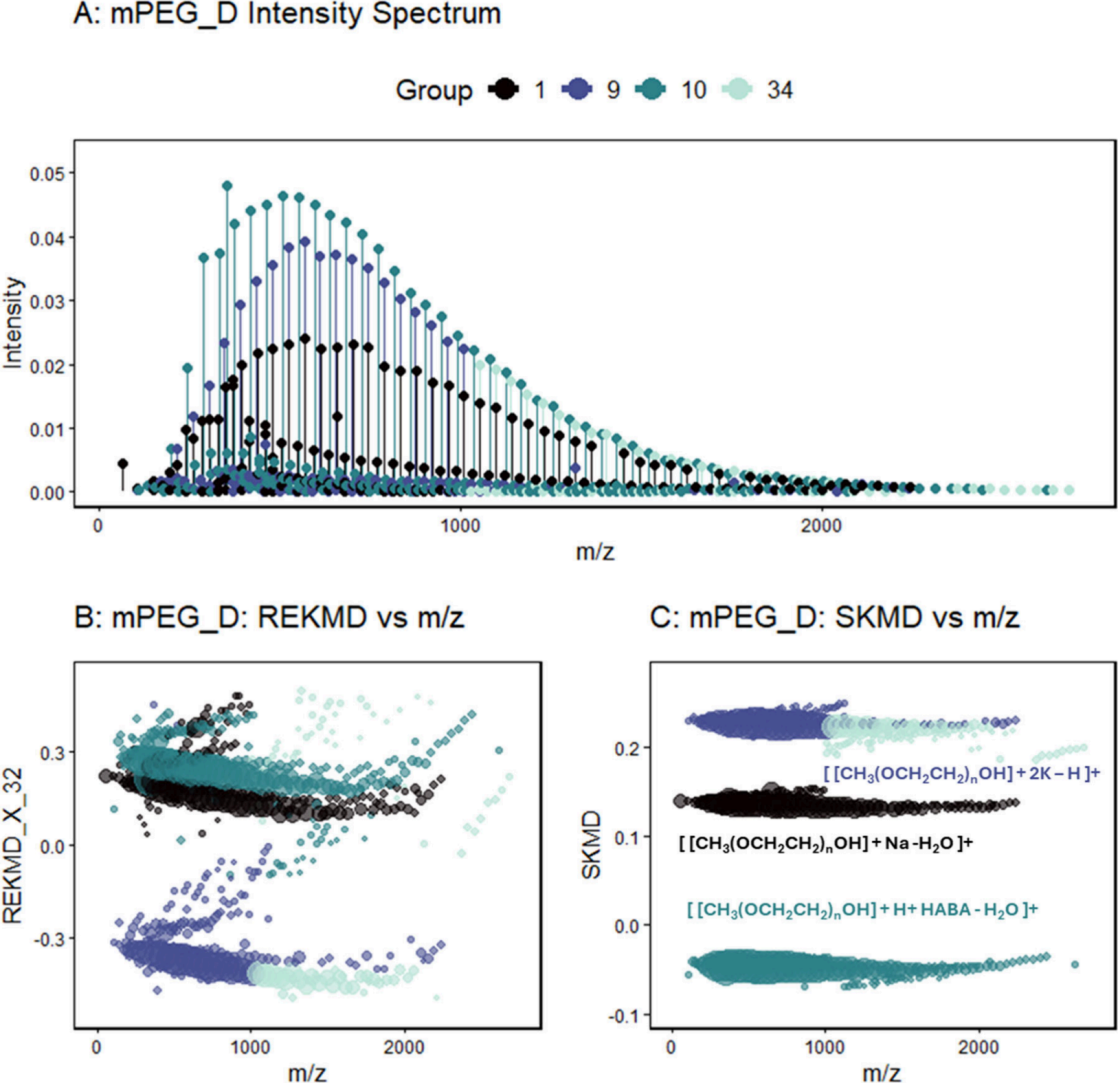
PEO-based peak series groupings via REKMD and SKMD plots of mPEG-D degraded supernatant spectra. (A) MS Spectra of ion peaks indicating the colored series represented in subsequent REKMD and SKMD plots. (B) REKMD (*X* = 34) plot illustrating the alignment of 44 Da spaced peaks, where consistent gradients identify related polymer series within the degraded mixture. (C) SKMD (*X* = 1) plot, which clusters these points more distinctly along the *y*-axis to reveal groupings with a more horizontal gradient. This optimized separation served as the primary basis for the automated grouping algorithm. In both plots, colors correspond to the assigned SKMD groups, while the *x*-axis (*m*/*z*) remains consistent to allow for direct comparison between the two transformation methods.

The observed spectra shifts (chain degradation) are corroborated in Figure 9 heatmaps, illustrating a clear migration from mPEG high-order oligomers (*n* > 30) to PEO truncated fragments (*n* < 20) following treatment. This *M*_w_ reduction is accompanied by a diversification of the adduct landscape; while the control is dominated by matrix-derived HABA complexes, the degraded samples exhibit a higher relative abundance of lower-mass ’nonstandard’ ions. This shift likely reflects the increased accessibility of terminal functional groups in shorter chains to enzymatic oxidation and subsequent ether bond cleavage.

**Figure 9:**
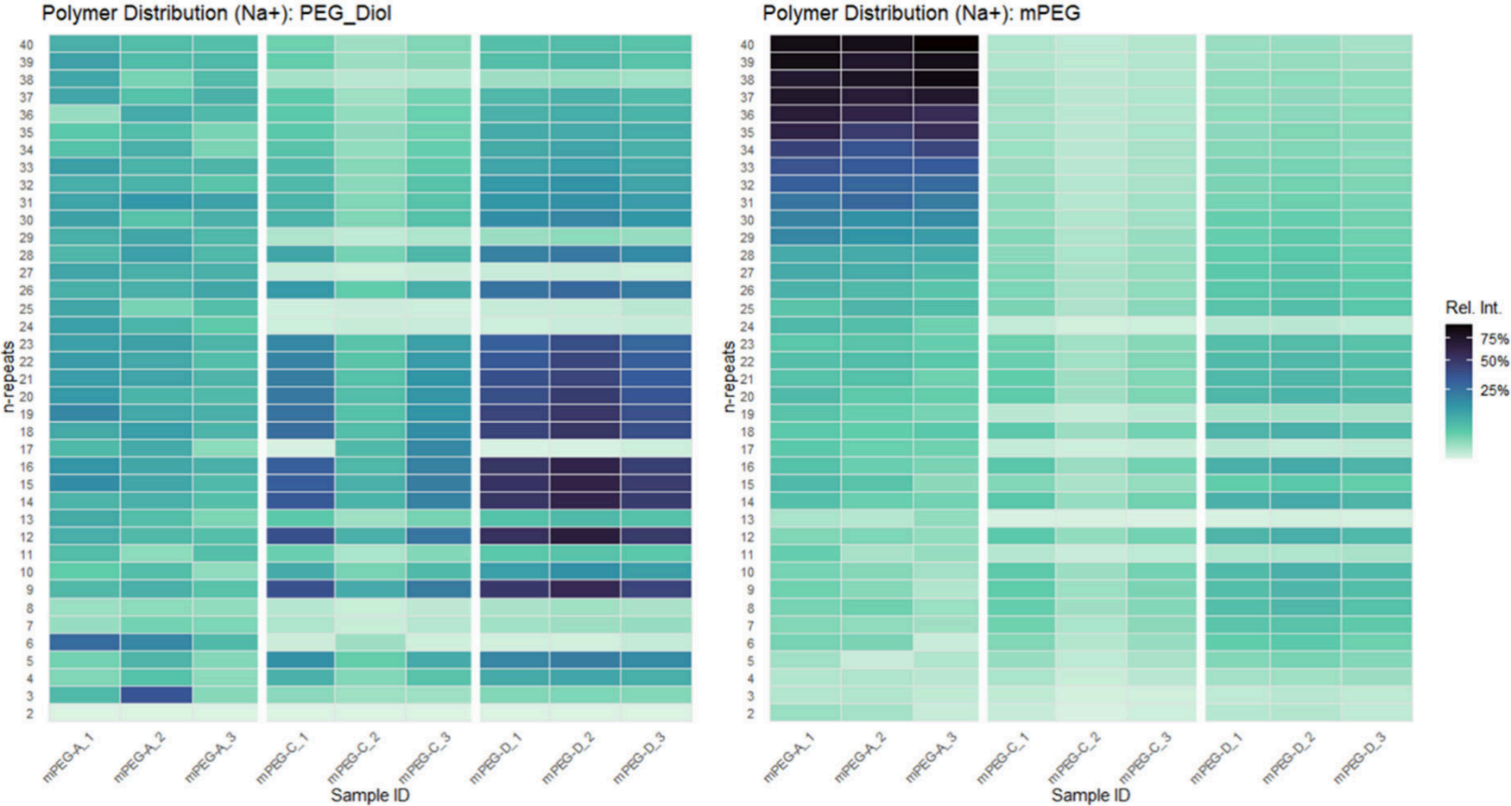
Heatmap distribution of PEO diol and mPEG oligomers by repeating *n*-mer units. Peak intensities were identified across sample triplicates (bottom axis) for untreated mPEG in THF (mPEG-A), the sterile Bushnell-Haas medium control (mPEG-C), and the inoculated microbial degradation sample (mPEG-D).

Smaller metabolite intensity inspection depicted in Figure 10 and Figure 11. The localized relative intensity heatmap of small metabolites (Figure 10) indicates that untreated control spectra (mPEG-A) exhibit trace signals for 2-hydroxyacetic acid and acetaldehyde when viewed on a local relative intensity scale. While other identified metabolites appear at even lower levels, a comparison with samples C and D (Figure 11) confirms that these mPEG-A peaks reside within the baseline noise. Consequently, these signals are interpreted as false positives; although they may stem from minor decarboxylation or isobaric interference, their minimal intensity suggests a negligible presence in the initial sample. This distinction underscores the importance of the filtering workflow in separating significant metabolic ″hits″ from stochastic background noise

**Figure 10:**
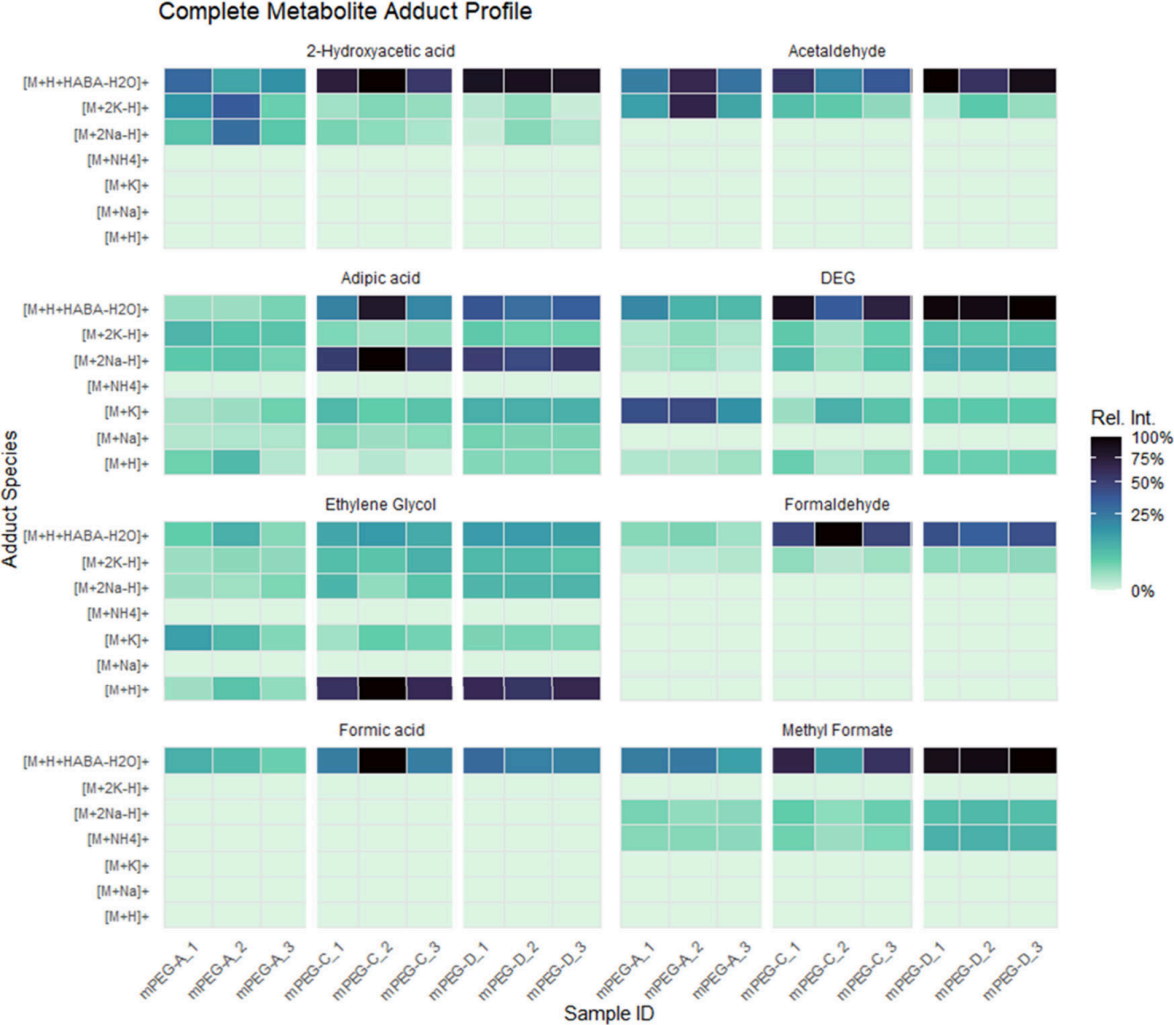
Local relative intensity heatmaps of PEG metabolite adduct species identified via MALDI-TOF MS. Individual panels represent probable degradation byproducts selected from literature to track specific polymer breakdown mechanisms. Each heatmap displays local relative abundance across sample triplicates (bottom axis), highlighting the appearance and disappearance of various adduct forms. across the untreated mPEG in THF (mPEG-A), the sterile Bushnell-Haas medium control (mPEG-C), and the microbial degradation sample (mPEG-D).

**Figure 11:**
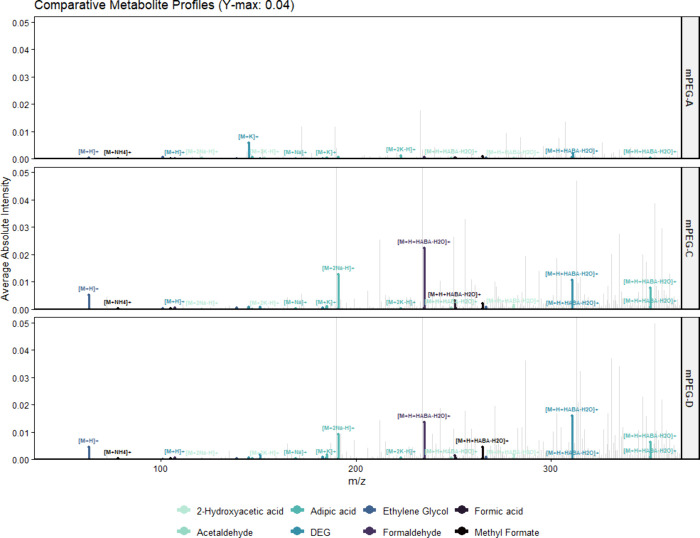
Comparative metabolite mass profiles (0–400 *m*/*z*) for mPEG-A, C, and D. The stacked absolute intensity plots illustrate the distribution of literature-matched metabolites (color-coded) against the spectral baseline. While untreated mPEG-A shows only trace, near-baseline signals for 2-hydroxyacetic acid and acetaldehyde, samples C and D exhibit clearly resolved peaks for key byproducts such as DEG, methyl formate, and ethylene glycol. The high degree of similarity between the BH blank and inoculated spectra suggests a significant contribution from abiotic degradation or media-specific interactions.

In contrast, Figure 10 reveals significantly stronger intensities in the control (mPEG-C) and inoculated (mPEG-D) columns, indicating the potential generation of these small molecules during the degradation process. Cross-referencing these results with Figure 11 reinforces this finding, as DEG, methyl formate, adipic acid, and ethylene glycol appear as distinct peaks clearly resolved above the baseline noise. Notably, the inoculated and control spectra display nearly identical high-intensity peak profiles, suggesting that the observed chemical changes may be driven by abiotic factors or specific media interactions common to both samples.

While a more focused analysis of the lower mass range is required to elucidate subtle sample variations, this heatmap demonstrates the capacity to detect polymeric compounds and small-molecule metabolites within a single data set.

The degradation of mPEG appears to be a multimodal process involving hydrolytic, thermal-oxidative, and microbial pathways. Hydrolysis is supported by literature suggesting the generation of metabolites such as adipic acid and diethylene glycol (DEG). Simultaneously, thermal-oxidative stress at 62 °C facilitates radical-mediated chain scission. As described by Grassie et al, radical attack on the polyether backbone generates alkoxy radicals that undergo beta-scission and hydrogen abstraction, yielding fragments such as methoxy acetaldehyde, formaldehyde, and low-molecular-weight hydrocarbons.^23^

Microbial action further accelerates this breakdown through extracellular enzymatic hydrolysis and radical production. Specifically, the mPEG degradation observed in Figure 11 likely follows a dehydrogenase-initiated pathway: the terminal hydroxyl group undergoes stepwise oxidation to an aldehyde and subsequently a carboxylic acid. This terminal acidification facilitates ether bond cleavage, yielding truncated mPEG chains and glyoxylic acid byproducts. Over time, the synergistic effect of enzymatic cleavage and radical-induced scission results in the significant reduction of *M*_w_ and *M*_n_ observed in the spectral data.

## Conclusions

This MALDI-TOF MS-based workflow, demonstrated using poly­(ethylene glycol) methyl ether, provides a framework for the untargeted analysis of polymer degradation in complex systems. By simultaneously monitoring polymeric decay and metabolite generation within a single sample, the method offers a comprehensive view of the biodegradation landscape. While default settings provide a functional baseline, optimization of each data set is recommended to ensure high-quality spectra and accurate interpretation. Such tailored adjustments mitigate spectral aliasing and improve data reliability, ultimately enabling a more precise investigation of metabolic byproducts in future polymer studies.

Future work should investigate optimization of matrix compositions for mixed biological/polymeric systems, including the evaluation of mixed MALDI matrices, which may offer improved compatibility across both polar and nonpolar analytes. Further studies should also include systematic evaluation of analytical sensitivity, repeatability. Additionally, the comparative assessment against alternative analytical workflows for polymer biodegradation products would also help further establish the robustness and broader applicability of the developed method.

Beyond PEG-based systems, this optimized MALDI-TOF MS and KMD workflow serves as a versatile template for investigating the degradation of a diverse range of synthetic and biobased polymers. The method is particularly well-suited for untargeted studies of complex environmental samples, such as microplastics in marine matrices or soil-bound agricultural films, where high biological background noise typically obscures chemical signals. By adjusting the Kendrick base unit to reflect different repeating motifs (e.g., CH_2_ for polyolefins or C_3_H_6_O for PPO), researchers can endeavor to isolate specific degradation pathways across varying chemical families.

## Supplementary Material



## Notes

Detailed methods for processing raw mass spectrometry data, implementation of the RANK1 function, and ion table generation for the analyzed adduct species (PDF)

## ∇

Royce Hub Building, The University of Manchester, Oxford Rd, Manchester M13 9PL, U.K
