# Supplementary material for: KMD-Enhanced MALDI-ToF MS for Polymer Biodegradation Analysis
Source: J Am Soc Mass Spectrom. 2026 Jul 14;37(8):2033–45. doi: 10.1021/jasms.6c00101 (PMC13450388; doi:10.1021/jasms.6c00101)
Supplement: Supplementary file 1 [file js6c00101_si_001.pdf]

Supporting Information

KMD-Enhanced MALDI-ToF MS for Polymer Biodegradation Analysis

AUTHOR NAMES

Sophia Y. van Mourik, Heather J. Walker, Anthony J. Ryan*

AUTHOR ADDRESS

Dainton Building, the University of Sheffield, Sheffield S3 7HF, UK

biOMICS Mass Spectrometry Facility, the University of Sheffield, Sheffield, S10 2TN

CORRESPONDING AUTHOR EMAIL ADDRESS

Sophia van Mourik (sophia.vanmourik@machester.ac.uk)

Raw Data Pre-processing

All the following is done via a custom R script available on request.

Transformation

A major source of spectral variance arises from differences in detected analyte intensity. For example, strong matrix peaks can obscure compounds of interest, particularly useful in small molecule analysis, so transformation can allow for more balanced and accurate peak interpretation. The two common transformation methods applied are the square root transformation, which is typically suitable for normally distributed data, and log transformation, which effectively reduces the impact of large outliers but can sometimes be overcorrect, introducing excessive noise into the data.

In polymer analysis, large peaks are typical, and applying a transformation often results in overcorrection (see Figure S1). The mPEG data appears to be skewed due to the higher peak density in the noise compared to the polymeric ions and matrix. Applying the transform only amplifies the noise in the spectra, rendering it ineffective. Consequently, it is recommended that the raw data remain untransformed; however, the transformation step is retained in the workflow to allow users the flexibility to customize processing based on their specific dataset.

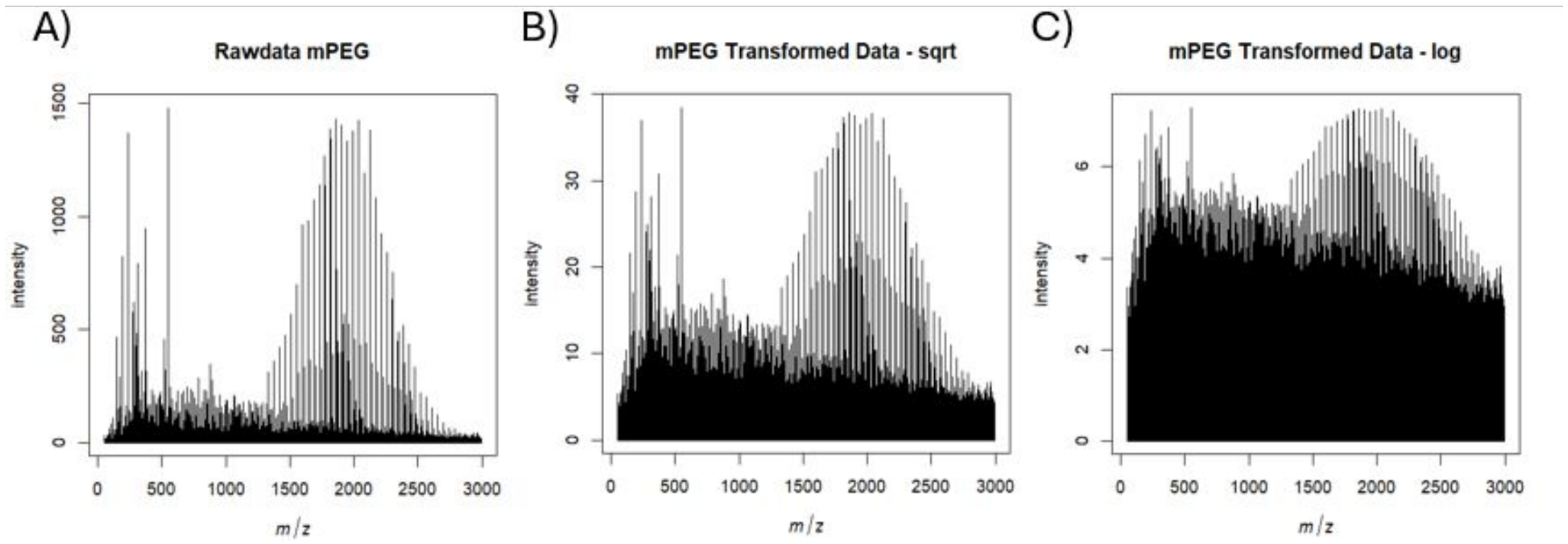

Figure S1. Impact of Transformation Methods on Raw MALDI-ToF-MS Spectra. (A) Untransformed Spectrum: Demonstrates a clear distribution of polymer ions well separated from matrix peaks and above the baseline noise.(B) Square-Root Transformation: Shows an increased signal-to-noise ratio (S/N) in the spectrum, but with some evidence of overcorrection.(C) Logarithmic Transformation: Exhibits significant overcorrection of the spectral data, making it difficult to distinguish between noise and polymer ion signals.

Smoothing

Spectral noise can obscure compound peak identification, making smoothing an important step to enhance clarity. However, excessive smoothing can merge closely spaced peaks, reducing resolution. Two common smoothing methods are used: the Savitzky-Golay filter, which applies a polynomial fit over a broader data range to preserve peak shape and provide higher accuracy, and the moving-average method, a simpler approach that uses adjacent data points to reduce noise but can distort peak shapes and decrease interpretive precision.

An important parameter in spectral smoothing is the Half Window Size (HWS), which determines the number of data points included in the smoothing calculation. Larger HWS values enhance noise reduction by increasing smoothing but also raise the risk of peak distortion.

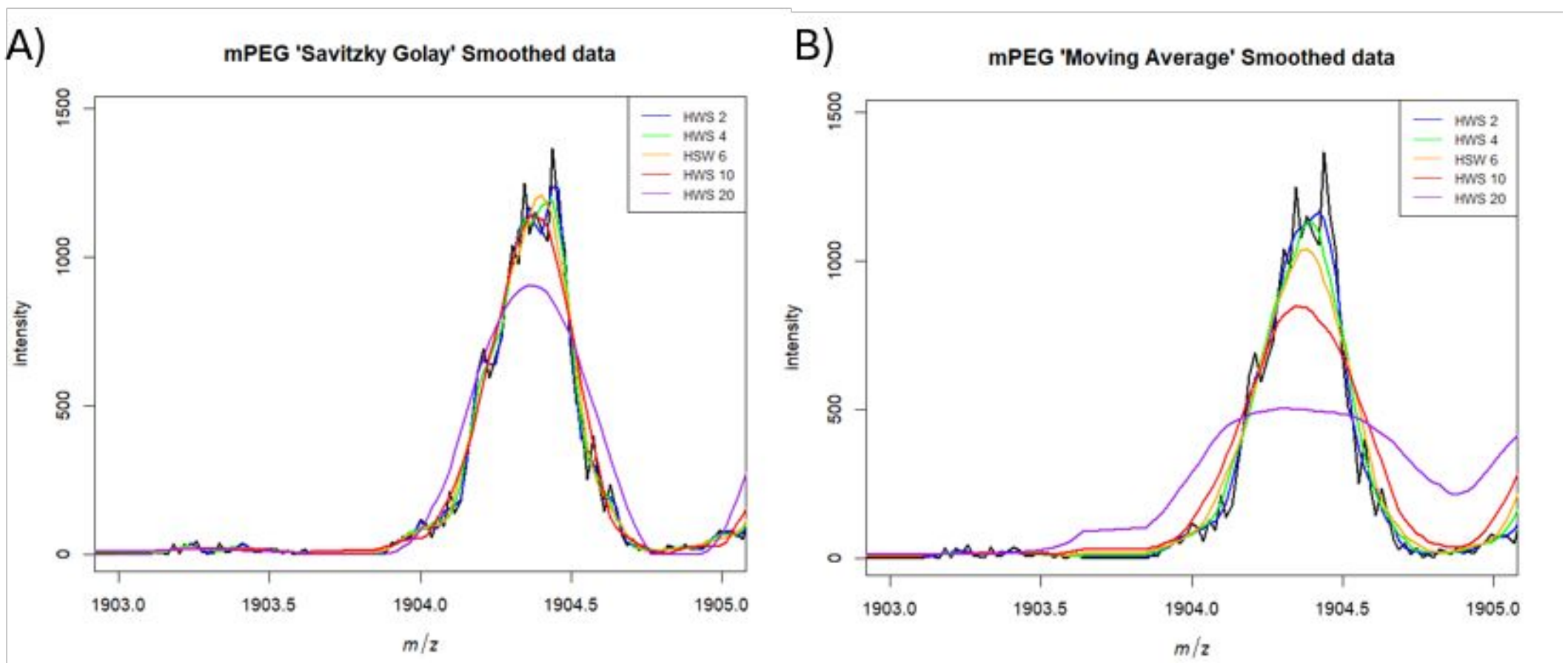

Figure S2 compares spectra smoothed with varying HWS values using both Savitzky-Golay and Moving Average methods. The Savitsky-Golay, at HWS 4 provided the best balance between peak shape preservation and signal intensity.

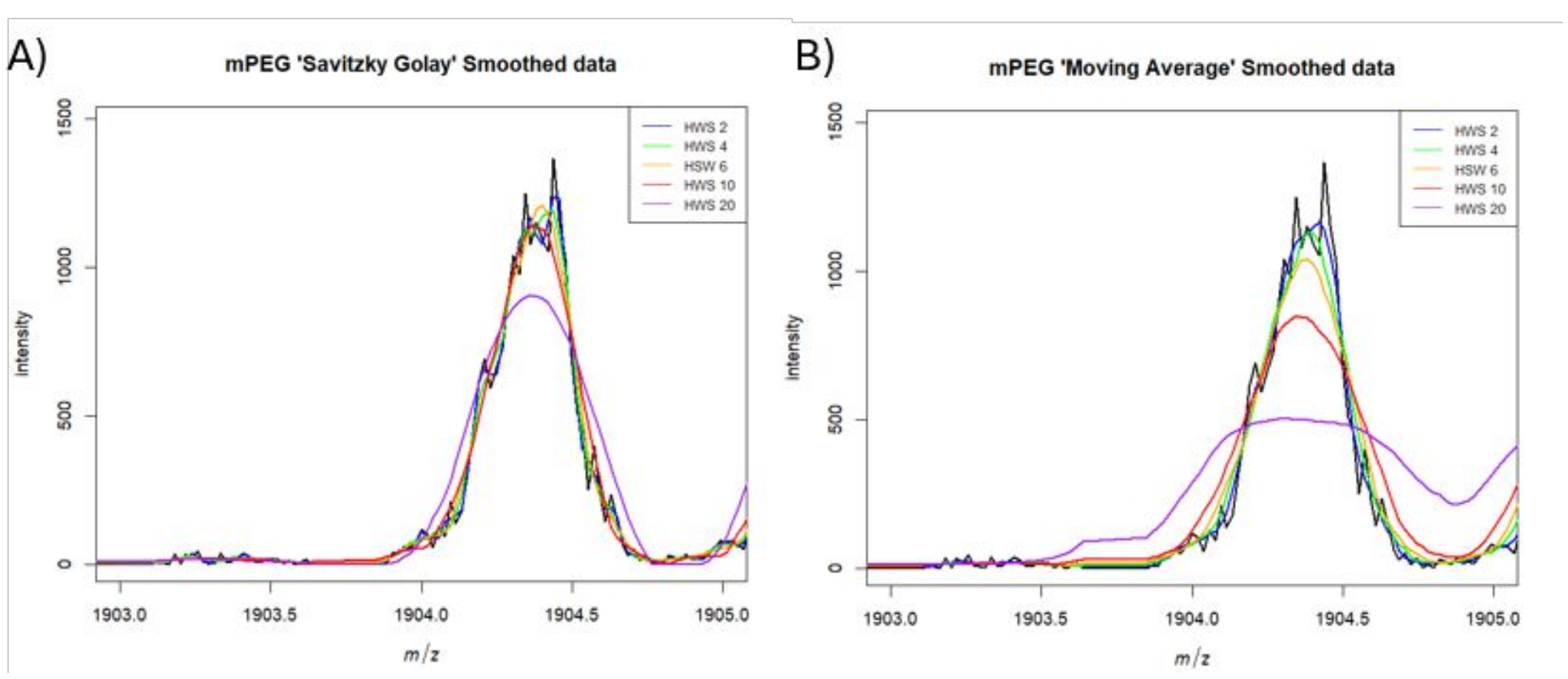

Figure S2. Comparison of Half Window Sizes (HWS) for (A) Savitzky–Golay and (B) Moving Average smoothing on a single peak. The lower HWS values and Savitsky–Golay was shown preserve peak shape and intensity most effectively.

Baseline correction

Baseline correction enhances the visibility of true peaks, removes false signals caused by noise, and improves peak intensity accuracy by adjusting for elevated baselines. The MALDIquant package offers four baseline correction methods to achieve these improvements.

The most applied method for MALDI ToF MS data is Statistics-sensitive Non-linear Iterative Peak-clipping algorithm (SNIP). It is considered the most effective method for preserving peak integrity, especially for complex baseline variations that occur in this type of raw data acquisition. It is using a threshold, slightly above the noise ratio of the data, to remove baseline noise.

The ConvexHull method flattens consistent baseline shifts by removing the convex envelope, producing a cleaner peak representation. TopHat and Median approaches operate by applying a moving average and removing median values, respectively. While all three effectively correct baseline drift and reduce noise, they can introduce artifacts, particularly in spectra with complex baseline features.

When using SNIP or TopHat baseline correction, the smoothing extent is controlled by the number of iterations (SNIP) or the Half Window Size (Top Hat), which define how many data points are included in the calculation. Increasing these parameters enhances noise reduction but can cause

over-smoothing. For high-resolution data, smaller values are recommended to preserve fine spectral details.

It is important to inspect both the full spectrum and individual peak smoothing in your data.

Figure **S3** shows that the SNIP, TopHat, and ConvexHull methods effectively flatten the baseline and reduce noise, whereas the Median correction accounts for polymer distribution, preserving the n-mer isotopic pattern. Over smoothing can be seen particularly in the single peak inspection. For this analysis, SNIP with 250 iterations was identified as the most effective baseline correction method for the dataset and recommended as the default for this type of data.

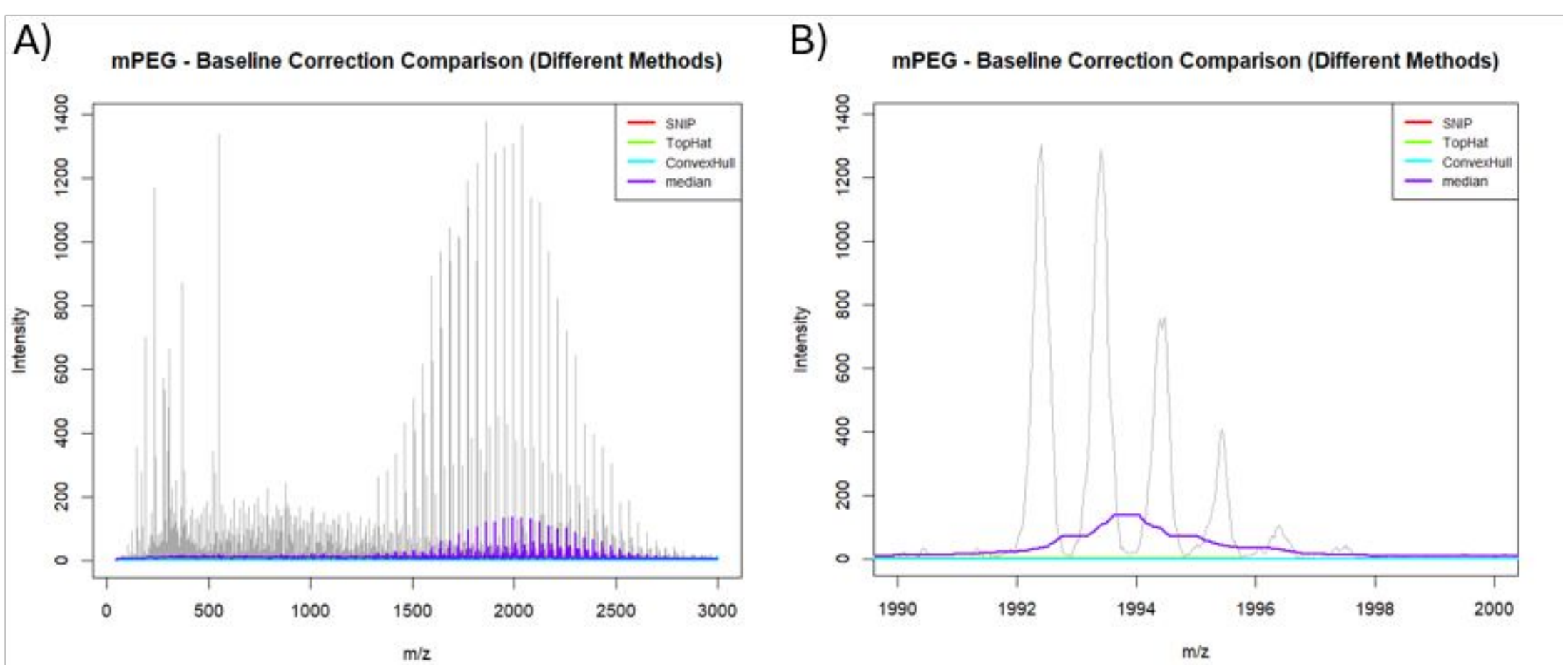

Figure S3. Comparison of Baseline Correction Methods on MALDI-ToF-MS Spectra. (A) Full Spectrum (Top figure) shows the overall baseline being calculated based on the method. (B) Single peak smoothing (Bottom figure) shows the effect of the baseline method on a set of peaks that are

neighboring. In both plots the media baseline correction is resulting in an overcorrection whilst the other three produce the same flat baseline.

Normalisation/Calibration

Intensity variability arises from complex ionization patterns influenced by factors such as analyte concentration, matrix effects, and instrumental variations. Therefore, signal intensity can be arbitrary; normalizing the data corrects for these biases and ensures accurate comparisons between samples.

Three methods are available to use within the specified MALDIquant packages. Total Ion Current (TIC) expresses relative individual peak intensity as a percentage of the overall spectrum and is often the default method applied. A more rigorous and computationally expensive method is Probabilistic Quotient Normalization (PQN). It requires additional calculations to scale each peak relative to its precursor ion. While this approach improves accuracy by reducing over or under representation of compounds, it is less efficient for large datasets and demands substantially more computational resources. Lastly, median normalization can be used in cases of particularly noise or irregular data values. As per its namesake, it uses the median to normalize values and is less sensitive to extreme values or outliers so is more robust to prevent outliers from disproportionately influencing the normalization process.

Peak Alignment: Warping

Peak alignment is performed to compensate for fine mass discrepancies between m/z values of samples that arise from instrument detection drift over time. The effect can be seen in **Error! Reference source not found.**, where warping has ensured that all samples are aligned. Aligned peaks allows for m/z value comparison across samples.

Warping or phase correction is performed using the Locally Weighted Scatterplot Smoothing (LOWESS) method, a locally weighted regression technique that adjusts for local peak shifts and is well suited to non-linear or localized distortions. Key parameters include the Half Window Size (HWS), which defines the number of points included in each peak; the Signal-to-Noise Ratio (SNR), which sets the threshold distinguishing peaks from background noise; and tolerance, which determines how similar two peaks must be to be considered identical. Increasing tolerance groups more peaks together, potentially enhancing overall signal intensity.

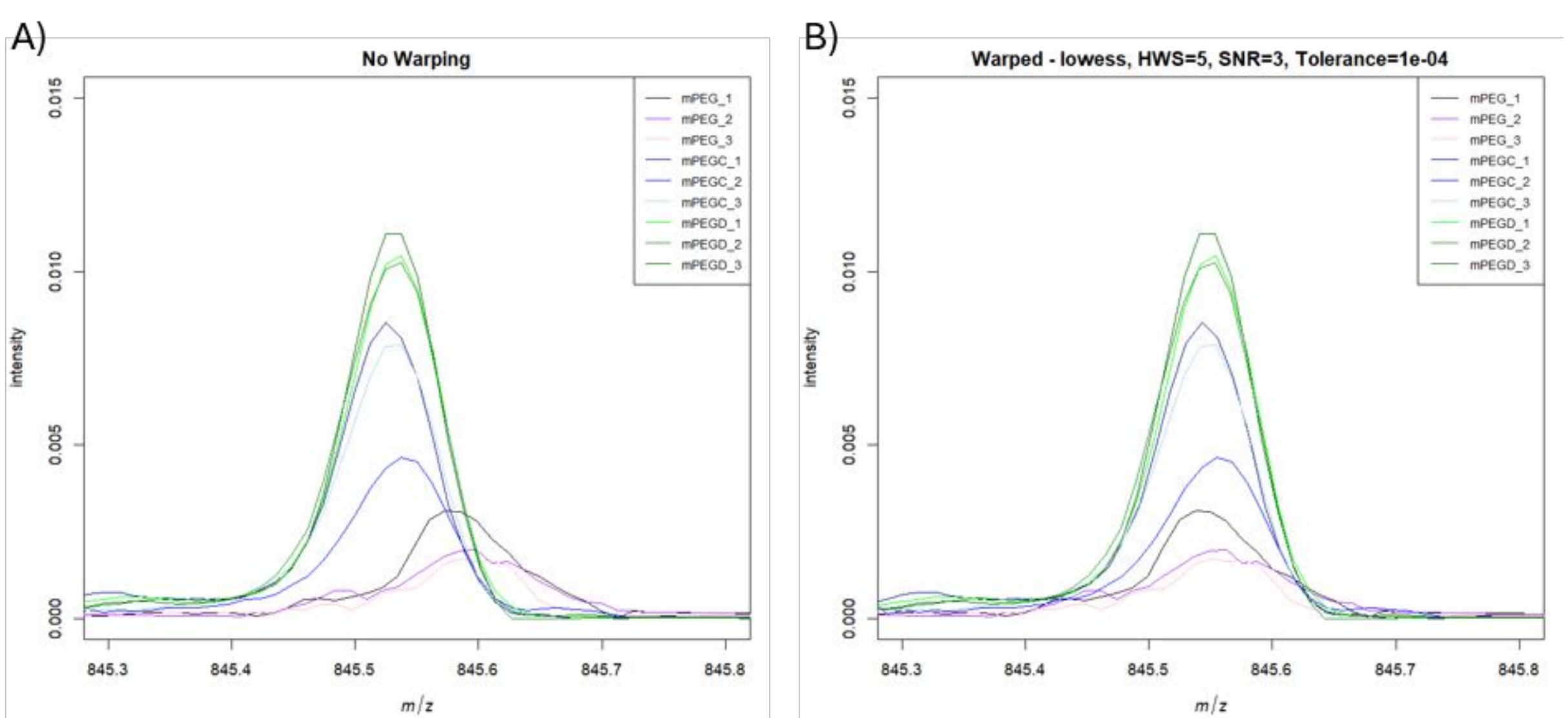

Figure S4. Effect of warping/phase correction on a single peak from the mPEG dataset. Each line represents an individual sample, showing (A) the peak drift present in non-warped data and (B) the improved alignment achieved after warping correction.

Peak detection & binning

MS data, is initially recorded as a ion cluster across a range of mass-to-charge (m/z) values and is then centroided into a single discrete m/z value during the peak detection step as shown in Figure S5. The choice of peak detection method strongly affects resolution, allowing adjustment between high and low settings based on analytical needs. Mean Absolute Deviation (MAD) is recommended for its robustness to outliers, defining noise thresholds using the data median. Alternatively, Friedman's Super Smoother uses moving averages to distinguish peaks from noise and performs better with broad peaks but is less resistant to outliers.

As with previous steps, HWS and SNR settings must be carefully considered. Additionally, the refineMZ and signal percentage parameters in the MALDIquant package influence peak identification and m/z calculation. The refineMZ method defines how peaks are detected, while the signal percentage (typically set to 50%) determines how much of the peak area contributes to the final m/z value.

Figure S5 illustrates how small discrepancies of less than 0.1 m/z can cause peaks to be detected separately. The peak binning step resolves these differences by unifying similar m/z values across all samples, enabling consistent comparison in downstream analyses. Strict binning prevents

multiple peaks from a single sample from occupying the same bin, producing more distinct peaks and suiting high-resolution data. In contrast, relaxed binning allows multiple peaks within a bin, better capturing broader peaks in lower-resolution datasets. The binning tolerance defines bin size, where smaller values create more bins. Accurate binning is essential for reliable peak intensity comparison in downstream analyses such as PCA.

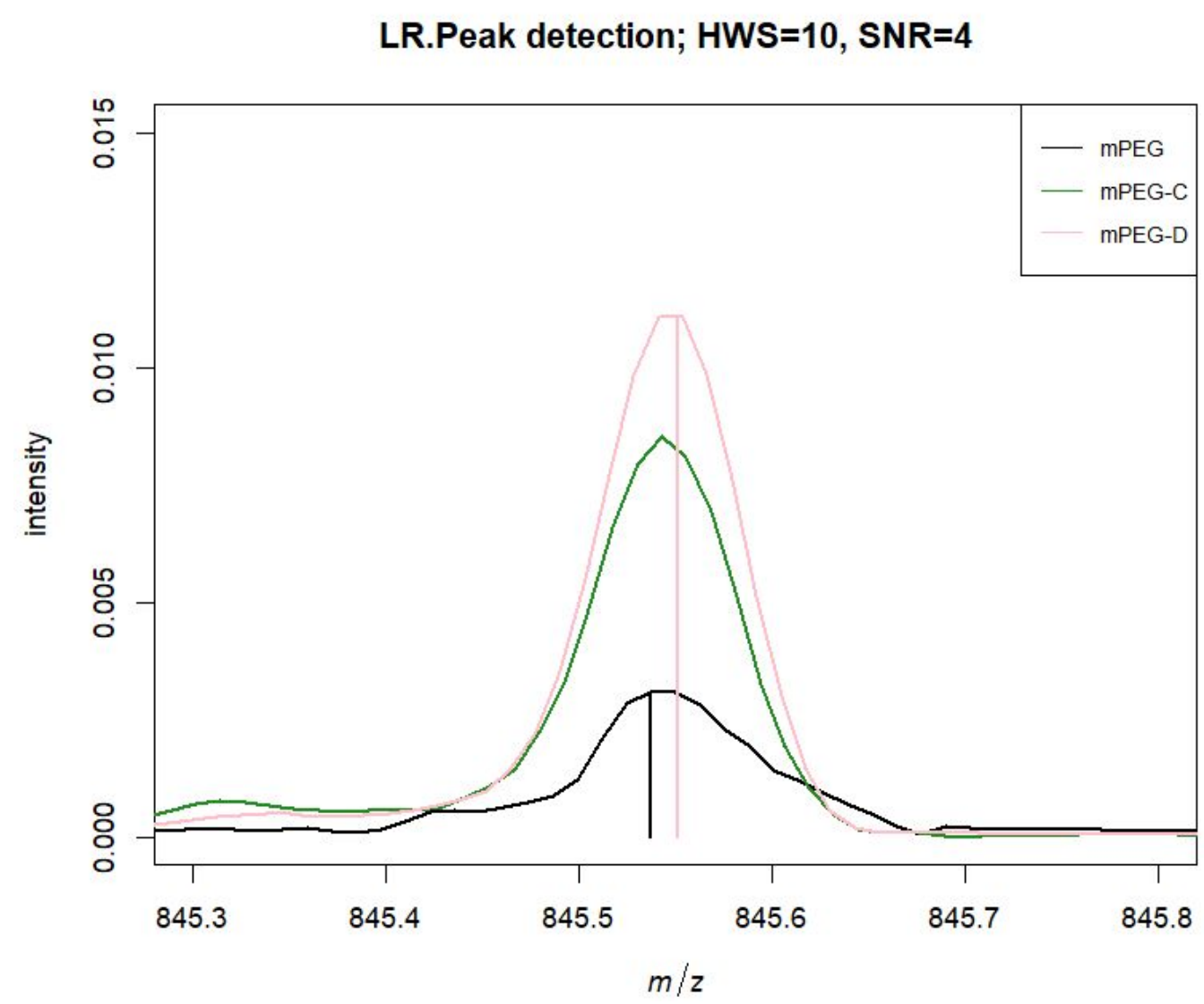

Figure S5. Peak Detection effect on three separate mPEG samples across a single peak. Illustrates that mPEG has detected slightly different peak compared to mPEG-D and mPEG-C despite the peak maximums being aligned.

Normalization Comparison – Linear Regression

Integrated analysis of the slope (LINEST) and R^2 values reveals variability across the sample groups; however, TIC normalization emerges as the most consistent and high-performing method

overall, appearing as the top performer in two out of the three samples (Figure S6, bar plots). This variability between samples is unsurprising given the markedly different treatment conditions applied to each. In some cases, alternative normalization methods resulted in slightly lower slope and R^2 values.

To minimise the risk of over-correction while still enabling a reliable normalization step for downstream processing, TIC performed best overall, yielding acceptable slope behaviour in mPEG-A and superior performance in both mPEG-C and mPEG-D. Importantly, all normalization methods maintained high R^2 values (>0.97) in the scatter plot comparisons.

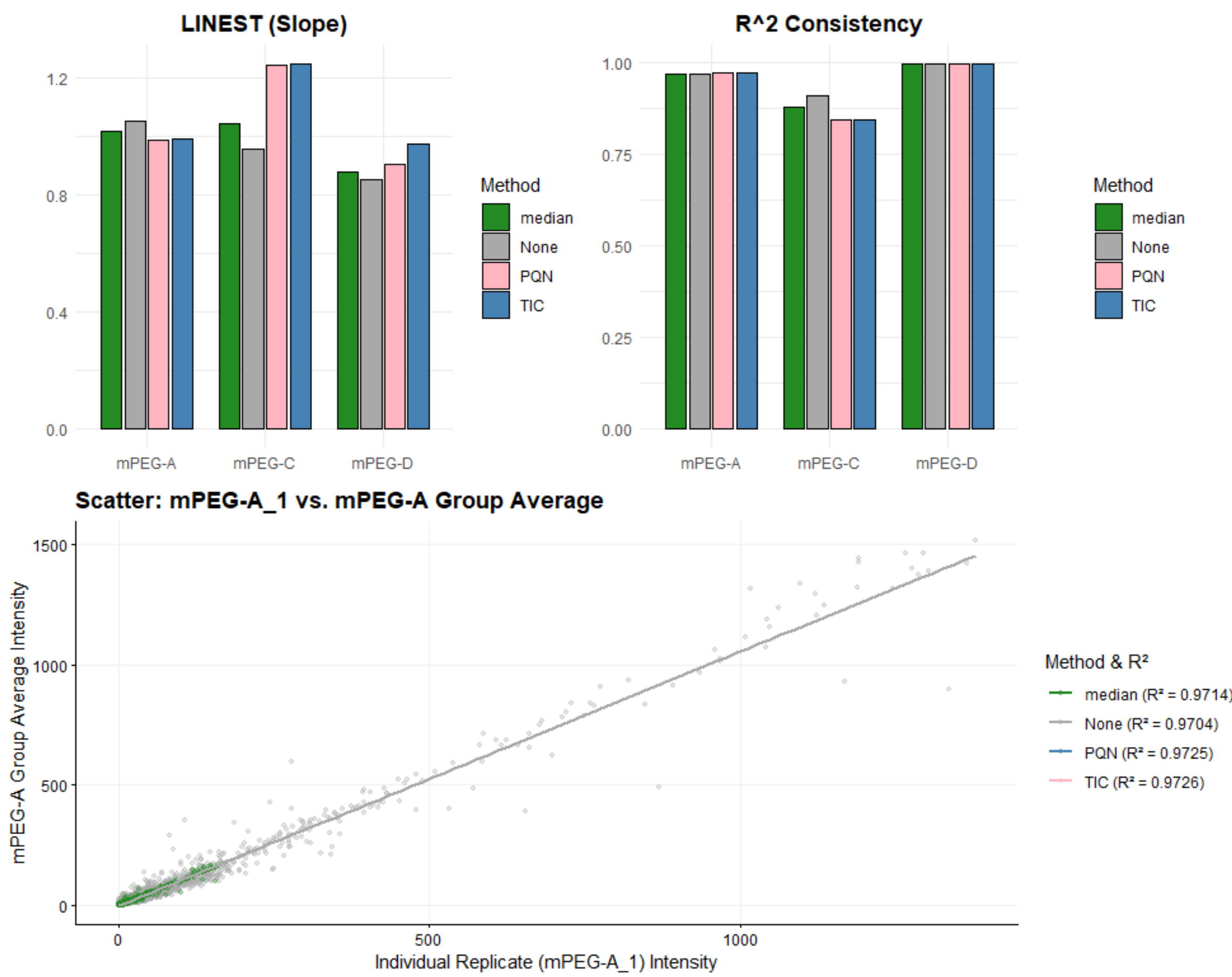

Figure S6. Linear Regression results to compare normalization efficacy

Kendrick Mass Defect and Ion Table Generation

The RANK1 function (equation%1) was applied to visualize and identify suitable X-divisor values for ion separation within the REKMD plots. Among the tested values, an X-divisors that generate a RANK1 value below 1%×%10^{-3} provided the most effective separation of adjacent ion series as shown in Figure S7 and was therefore selected for downstream analysis. The corresponding

REKMD coordinates generated using this divisor were subsequently extracted from the plot and incorporated as calculated columns within the R script for further processing.

$$\mathrm{RANK1(R1,R2)} = |\mathrm{KMD}_{+\mathrm{R2}}(\mathrm{R1,X})| \quad (1)$$

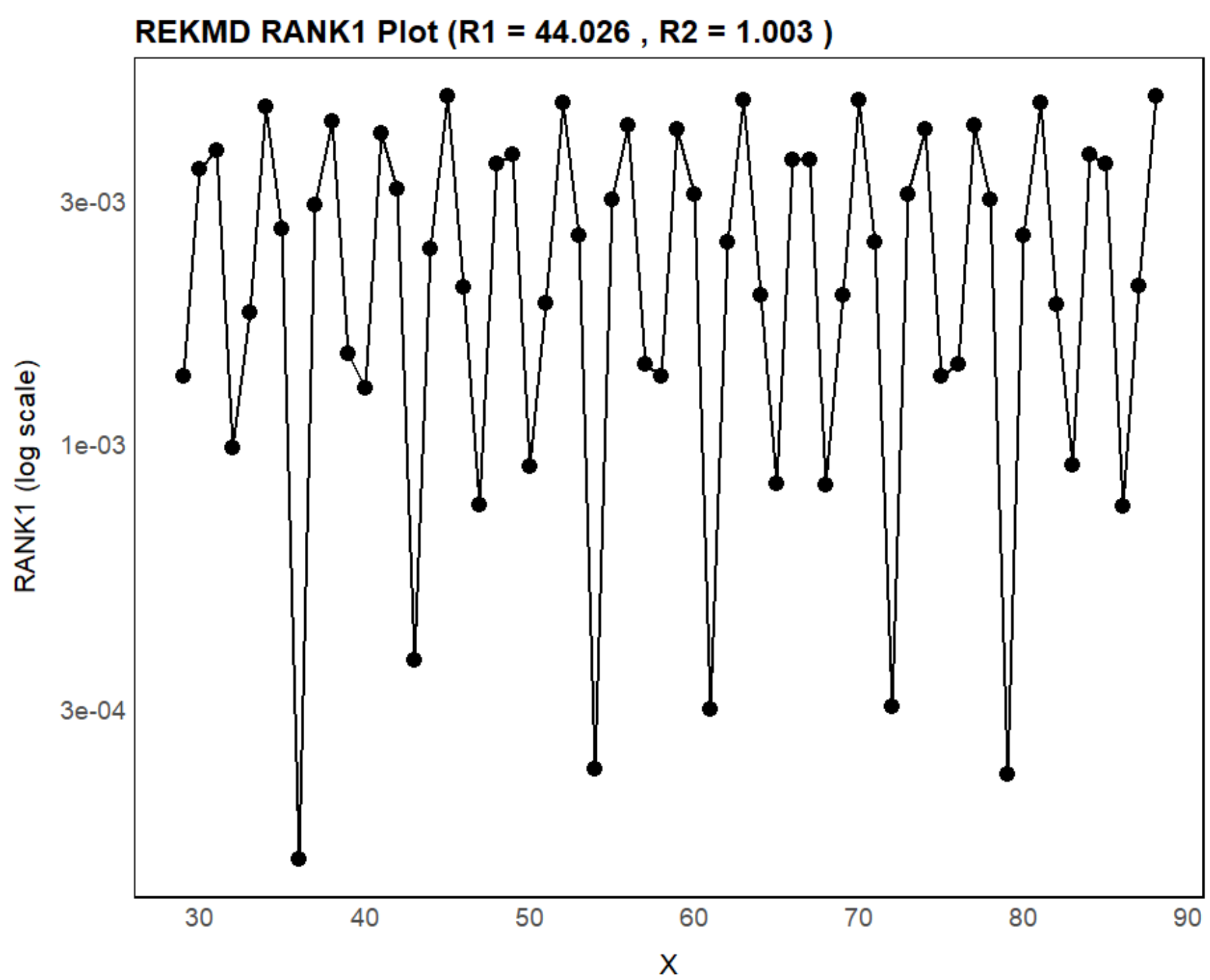

Figure S7. RANK-1 plot on a logarithmic scale using R_1%=%44.026 and R_2%=%1.003 to assess X-divisor performance for ion separation in the REKMD analysis of the PEO backbone. Points with RANK-1 values below 1%×%10^{-3} indicate substantially improved separation of ion families,

and these minima were subsequently used to select optimal X-divisors for downstream REKMD calculations in the R workflow.

Common Adduct Formations

To enable accurate construction of the heatmaps, a comprehensive list of ion adducts was assembled and used in the analysis (Table%S1). This list includes all singly and multiply charged species incorporated into the workflow, along with each adduct's standard notation, charge-multiplier value used for mass normalization, and the corresponding exact mass shift (Da) applied during adduct assignment. The table integrates both commonly observed adducts and additional HABA-derived species, ensuring robust annotation of the complex adduct patterns characteristic of PEG-based spectra.

Table S1. Complete Ion Adduct List Used for Heatmap Calculations. Where [M] is the fragment of interest.

Ion Name	Charge Multiplier	Mass Shift (Da)
$[M+3H]^{3+}$	0.33	1.00728
$[M+H+2Na]^{3+}$	0.33	8.33459
$[M+2H+Na]^{3+}$	0.33	15.6625

$[M+3Na]^{3+}$	0.33	22.9892
$[M+2H]^{2+}$	0.5	1.00728
$[M+H+NH_4]^{2+}$	0.5	9.52055
$[M+H+Na]^{2+}$	0.5	11.9982
$[M+H+K]^{2+}$	0.5	19.9852
$[M+ACN+2H]^{2+}$	0.5	21.5206
$[M+2Na]^{2+}$	0.5	22.9892
$[M+2ACN+2H]^{2+}$	0.5	42.0338
$[M+3ACN+2H]^{2+}$	0.5	62.5471
$[M+H]^{+}$	1	1.00728
$[M+Na]^{+}$	1	22.9892
$[M+Na–H_2O]^{+}$	1	4.97865
$[M+2Na–H]^{+}$	1	44.9712
$[M+K]^{+}$	1	38.9632
$[M+K–H_2O]^{+}$	1	20.9526
$[M+2K–H]^{+}$	1	76.919
$[M+NH_4]^{+}$	1	18.0338
$[M+NH_4–H_2O]^{+}$	1	0.02326
$[M+CH_3OH+H]^{+}$	1	33.0335
$[M+ACN+H]^{+}$	1	42.0338

$[M+ACN+Na]^+$	1	64.0158
$[M+2ACN+H]^+$	1	83.0604
$[M+IsoProp+H]^+$	1	61.0653
[M+IsoProp+Na+H]	1	84.0551
$[M+DMSO+H]^+$	1	79.0212
$[M+H+HABA–H_2O]^+$	1	225.066
$[M+H+2HABA–2H_2O]^+$	1	449.114
$[M+H+HABA–H_2O+Na]^+$	1	248.055
$[M+H+HABA–H_2O+K]^+$	1	264.029
$[2M+H]^+$	2	1.00728
$[2M+NH_4]^+$	2	18.0338
$[2M+Na]^+$	2	22.9892
$[2M+K]^+$	2	38.9632
$[2M+ACN+H]^+$	2	42.0338
$[2M+ACN+Na]^+$	2	64.0158
